# Potential Implications of Body Mass Composition Changes in Heart Failure Patients in the Era of SGLT2i, GLP-1 RA, and GIP/GLP-1 RA

**DOI:** 10.3390/ph18111726

**Published:** 2025-11-13

**Authors:** Katarzyna Gryglewska-Wawrzak, Agnieszka Kapłon-Cieślicka, Agnieszka Pawlak, Anna Tomaszuk-Kazberuk, Paweł Rubiś, Jacek Niedziela, Agata Bielecka-Dąbrowa

**Affiliations:** 1 Department of Cardiology and Congenital Diseases of Adults, Polish Mother’s Memorial Hospital Research Institute, 93-338 Lodz, Poland; 2 First Department of Cardiology, Medical University of Warsaw, 02-091 Warsaw, Poland; 3 Department of Cardiology, National Medical Institute of the Ministry of the Interior and Administration, 02-507 Warsaw, Poland; 4 Department of Cardiology, Lipidology and Internal Medicine with Intensive Coronary Care Unit, Medical University of Bialystok, 15-089 Białystok, Poland; 5 Clinical Department of Cardiac and Vascular Diseases, Institute of Cardiology, Jagiellonian University Collegium Medicum, John Paul II Hospital, 31-202 Krakow, Poland; 6 Department of Cardiology, Heart Failure and Rehabilitation, Andrzej Frycz Modrzewski Krakow University, 30-705 Krakow, Poland; 7 Department of Cardiac Rehabilitation, Uzdrowisko Ustron, American Heart of Poland, 43-450 Ustron, Poland; 8 Department of Preventive Cardiology and Lipidology, Medical University of Lodz, 90-419 Lodz, Poland

**Keywords:** obesity, heart failure, body mass composition, sarcopenia

## Abstract

Obesity is a complex, multifactorial disease wherein the excessive accumulation of adipose tissue leads to adverse health outcomes, such as diabetes, cardiovascular disease and musculoskeletal disorders. Obesity also impacts both the risk and the clinical prognosis of heart failure (HF). The accumulation of adipose tissue results in metabolic dysregulation, including increased levels of pro-inflammatory cytokines and adipokines. These alterations are strongly associated with the development and progression of HF. Another significant comorbidity in patients with HF is sarcopenia, characterized by progressive loss of muscle mass and strength, affecting the quality of life. The study aims to critically synthesize the mechanisms by which modern pharmacological treatments—sodium-glucose cotransporter-2 (SGLT2) inhibitors, glucagon-like peptide-1 receptor (GLP-1R) agonists, and dual GIPR/GLP-1R agonists—modulate body mass composition, and to analyze the specific implications of these changes (e.g., visceral fat reduction versus lean mass loss) for heart failure (HF) prognosis and management.

## 1. Methods

This article is a narrative review of the literature. A search was conducted using the PubMed, Scopus, and Google Scholar databases for relevant articles. Search terms included combinations of “heart failure,” “HFpEF,” “HFrEF,” “obesity,” “body composition,” “sarcopenia,” “SGLT2 inhibitors,” “GLP-1 receptor agonists,” and “GIP/GLP-1 receptor agonists.” We prioritized findings from recent (2020–2025) large-scale clinical trials (RCTs), meta-analyses, and comprehensive reviews, particularly those focused on body composition mechanisms and HF-specific outcomes rather than glycemic control alone.

## 2. Obesity and Heart Failure

The prevalence of obesity is increasing. Overweight and obesity are projected to affect nearly 3 billion adults (approx. 50% of the world’s adult population) by 2030. Of particular concern is the rising prevalence of obesity requiring medical intervention, which carries serious implications for health systems [1]. One of the cardiac complications of obesity is heart failure (HF), especially that with preserved left ventricular ejection fraction (HFpEF). Obesity-related factors are estimated to cause 11% and 14% of HF cases in men and women, respectively. The prevalence of HF is 2–3% of the population in industrialized countries [2].

The association between obesity and HF has become a focal point of cardiovascular research, particularly as the prevalence of obesity has escalated to epidemic proportions. Obesity significantly impacts both the risk and the clinical prognosis of HF. While obesity serves as a fundamental risk factor for the development of HF through various biological mechanisms, it paradoxically appears to confer certain survival advantages in specific patient populations, a phenomenon often referred to as the “obesity paradox” [3].

**Figure 1:**
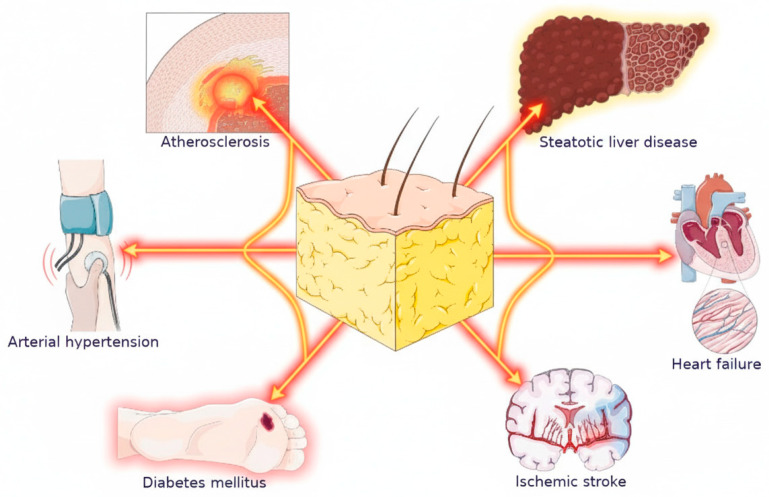
Adipose tissue as a central pathophysiological organ in the development of cardiometabolic diseases. The figure illustrates how dysfunctional, excess adipose tissue (center) is not a passive energy depot but an active endocrine organ. It secretes pro-inflammatory and pro-atherogenic mediators, directly driving the development of key comorbidities such as arterial hypertension, diabetes mellitus, atherosclerosis, and steatotic liver disease. From HF management perspective, these conditions are not merely comorbidities but are mechanistically linked by obesity. This highlights the implication that interventions targeting the reduction and functional improvement of adipose tissue (e.g., via novel metabolic therapies) are a critical component of comprehensive HF patient management. The figure was partly generated using Servier Medical Art (https://smart.servier.com), provided by Servier (Suresnes, France), licensed under a Creative Commons Attribution 4.0 International License (https://creativecommons.org/licenses/by/4.0/ (accessed on 5 November 2025)) and modified by the authors for the specific context of this review.

Obesity leads to a variety of pathological changes within the cardiovascular system, affecting both cardiac structure and function (Figure 1). The accumulation of adipose tissue results in metabolic dysregulation, characterized by increased levels of pro-inflammatory cytokines and adipokines. These alterations can exacerbate left ventricular hypertrophy, promote endothelial dysfunction, and lead to increased stiffness of the myocardium, which are pivotal changes that contribute to HF [4,5]. Additionally, various hormonal dysregulations, such as increased levels of leptin and decreased levels of adiponectin, are observed in obese patients, which have been linked to a propensity for HF development.

Other mechanisms include increased hemodynamic stress, particularly due to heightened blood volume and cardiac output requirements. This chronic overload results in left ventricular dilatation and subsequent systolic and diastolic dysfunction. Epidemiological studies have consistently documented that the risk of developing HF increases with each unit rise in body mass index (BMI), illustrating a direct relationship where a mere increase of one kg/m^2^ in BMI is associated with a greater risk of HF by approximately 5% in men and 7% in women [6].

Interestingly, research has emphasized the role of weight loss as a critical therapeutic target in managing obese patients with HF. Studies have demonstrated that intentional weight loss can improve cardiac function and overall clinical outcomes, potentially reducing hospitalization rates [7,8]. Bariatric surgery, in particular, has yielded notable success in reducing body mass and subsequent HF symptoms, suggesting a paradigm shift towards integrating obesity management with HF treatment strategies [9].

The obesity paradox, however, complicates this relationship. Some research indicates that obese patients with HF often experience better survival rates compared to their normal-weight counterparts, particularly within specific forms of HF, such as HFpEF [10,11]. This paradox is hypothesized to arise from the energy reserves that excess body fat provides, potentially allowing obese individuals to withstand the catabolic stress of advanced HF [12]. Additionally, this phenomenon may be influenced by hormonal and metabolic factors that vary between individuals [13].

Within this context, patients with obesity-related HFpEF exhibit distinct pathophysiological characteristics that can affect their management and outcomes. For instance, the interplay between BMI and exercise capacity reveals substantial differences in peak oxygen consumption rates among obese patients compared to non-obese individuals [14]. Furthermore, the assessment of biomarkers, such as N-terminal pro-B-type natriuretic peptide (NT-proBNP), highlights the challenges in accurately diagnosing and managing heart failure across different body habitus, owing to the paradoxically lower circulating peptide levels associated with adiposity [15].

Clinical guidelines now increasingly acknowledge obesity as a significant comorbidity in HF management. Treatment pathways necessitate personalized approaches that consider metabolic status, body composition, and concurrent health conditions to ensure optimal outcomes. In particular, emerging pharmacotherapeutics—such as sodium-glucose cotransporter-2 (SGLT2) inhibitors, glucagon-like peptide-1 receptor (GLP-1R) agonists, including dual agonists targeting both GLP-1 and glucose-dependent insulinotropic polypeptide (GIPR/GLP-1R)—may offer a dual benefit by managing diabetes and relieving HF symptoms in obese patients. While these agents were initially developed for glycemic control in type 2 diabetes mellitus (T2DM), their profound cardiovascular benefits have highlighted additional mechanisms of action. Indeed, a crucial pathophysiological link between obesity, T2DM, and HF is the chronic activation of systemic inflammation and oxidative stress. This heightened oxidative stress, resulting from mitochondrial dysfunction and excess reactive oxygen species (ROS) in adipose tissue and the myocardium, is a key driver of endothelial dysfunction, myocardial fibrosis, and subsequent cardiovascular complications. Therefore, a significant component of the therapeutic benefit of these modern agents is thought to stem from their ability to mitigate these deleterious pathways (including direct antioxidant and anti-inflammatory effects), representing an advantage beyond glycemic control alone [8].

## 3. Sarcopenia in Heart Failure

**Figure 2:**
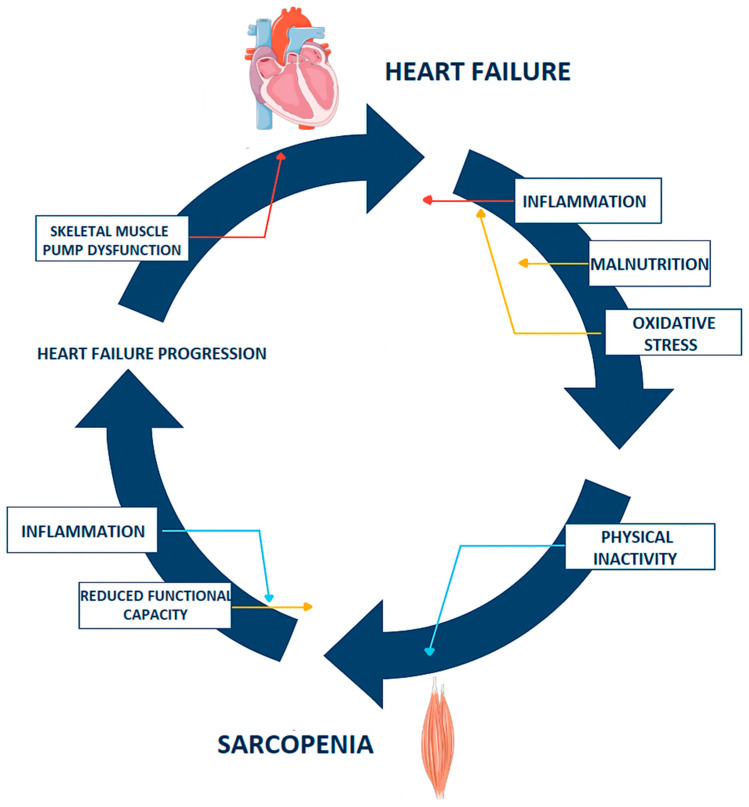
Mechanisms linking heart failure and sarcopenia. The figure was partly generated using Servier Medical Art (https://smart.servier.com), provided by Servier (Suresnes, France), licensed under a Creative Commons Attribution 4.0 International License (https://creativecommons.org/licenses/by/4.0/ (accessed on 5 November 2025)) and modified by the authors for the specific context of this review.

Body mass composition, particularly in the context of sarcopenia, is a critical aspect of HF management (Figure 2). Sarcopenia, which is characterized by the progressive loss of skeletal muscle strength or function and often accompanied by a loss of mass, is increasingly recognized as a significant comorbidity in patients with heart failure. This condition poses unique challenges due to its contributions to morbidity, mortality, and overall quality of life among HF patients [16,17].

Sarcopenia commonly occurs in HF patients due to a combination of factors, including systemic inflammation, physical inactivity resulting from disease symptoms, and the adverse effects of various treatments, such as diuretics [18]. The prevalence of sarcopenia in patients with HF varies widely, indicating that it is a significant concern in both heart failure with preserved ejection fraction (HFpEF) and reduced ejection fraction (HFrEF) [19,20]. Muscle loss associated with sarcopenia correlates with diminished exercise capacity, frailty, and an increased risk of hospitalizations and adverse outcomes [18].

A significant challenge is the diagnosis itself, particularly in HF populations susceptible to fluid retention, which can mask muscle loss. Clinical diagnosis, guided by standards such as the European Working Group on Sarcopenia in Older People (EWGSOP2), relies on assessing three key domains: (1) low muscle strength, (2) low muscle quantity/quality, and (3) low physical performance [21]. The applicability of these methods in HF is summarized in Table 1.

Furthermore, the clinical picture is often complicated by sex-related variations. While men typically have higher baseline muscle mass, post-menopausal women may experience accelerated muscle loss, potentially altering their risk profile and response to interventions [22]. An even greater challenge is sarcopenic obesity (SO), which presents a significant clinical problem that impacts medication efficacy. This condition is characterized by an increase in body fat and a corresponding decrease in muscle mass, affecting pharmacokinetics—specifically drug absorption, distribution, metabolism, and excretion. Understanding how sarcopenic obesity alters these processes is vital for optimizing medication regimens, particularly in elderly populations disproportionately affected by this condition [23].

The relationship between BMI and HF is complex. Increasing BMI is more strongly linked to the risk of developing HFpEF compared to HFrEF, underscoring the need for tailored interventions depending on body composition [20]. Additionally, loss of lean body mass exacerbates HF symptoms, as muscle strength and function are crucial for maintaining mobility and activity levels in this population [24]. Conversely, HF itself can also lead to alterations in body composition, with a redistribution of body fat, which complicates the management of both heart function and metabolic health [25]. The causes of sarcopenia in patients with HFrEF, HFmrEF, and HFpEF are summarized in Table 2 and Table 3.

## 4. Pharmacotherapy of Heart Failure in the Context of Obesity

This review argues that the new generation of metabolic drugs represents a paradigm shift in HF management, moving beyond symptom control and neurohormonal blockade towards directly targeting the metabolic and inflammatory drivers of HF, particularly HFpEF. We critically analyze how the quality of weight loss (i.e., preferential reduction in ectopic fat like visceral and epicardial adipose tissue) rather than the quantity of weight loss alone, mediates their cardiovascular benefits. This is not merely a problem of excess weight, but of metabolically active visceral and epicardial adipose tissue (EAT), which drives local inflammation, myocardial lipotoxicity, and oxidative stress, contributing directly to the pathophysiology of diastolic dysfunction and HFpEF [26].

The pharmacotherapy of HF primarily relies on a combination of medications that address different aspects of the condition, often referred to as the “four pillars” for HFrEF: angiotensin-converting enzyme (ACE) inhibitors or angiotensin receptor-neprilysin inhibitors (ARNIs), beta-blockers, mineralocorticoid receptor antagonists (MRAs), and sodium-glucose cotransporter-2 (SGLT2) inhibitors. Diuretics are also used for symptom management, particularly in patients with volume overload. The pharmacotherapy of HFpEF primarily focuses on symptom management, particularly addressing volume overload with diuretics, and optimizing treatment for comorbidities, with SGLT2 inhibitors now established as a recommended first-line therapy to reduce cardiovascular events [27].

Beta-blockers used in HF may increase body weight, but their beneficial effect in patients with HFrEF has been confirmed. Although renin–angiotensin–aldosterone system (RAAs) inhibitors are not drugs dedicated to the treatment of obesity, some studies indicate that these medications can lead to modest weight loss or reduced weight gain in certain individuals, potentially by affecting leptin levels responsible for appetite or metabolism. RAAs inhibitors can have anti-inflammatory properties, which may play a role in preventing or mitigating obesity-related inflammation and its impact on metabolism [28]. While MRAs like spironolactone, eplerenone, and finerenone are primarily used for cardiovascular conditions such as HF and hypertension, their potential in managing metabolic derangements associated with obesity is an active area of research. MRAs have shown potential in influencing body mass, particularly in preventing weight gain and reducing visceral fat and are being investigated for their role in managing obesity-related metabolic complications by preventing white adipose tissue dysfunction and expansion, inducing the “browning” of white adipose tissue in animal models, a process that can increase energy expenditure and potentially reduce fat mass by increasing uncoupling protein 1 (UCP1) levels [29].

Emerging treatment strategies, including SGLT2 inhibitors, glucagon-like peptide-1 receptor (GLP-1R) agonists, and dual GLP-1/gastric inhibitory polypeptide (GIP) receptor agonists, play a pivotal role not only in glycemic control but also in improving cardiovascular outcomes, body mass management, and functional capacity in these patients. Integrating these therapies into comprehensive HF care may enhance symptom control, quality of life, and long-term prognosis, particularly in patients with obesity or metabolic dysfunction.

## 5. Sodium-Glucose Transport Protein 2 (SGLT2) Inhibitors

SGLT2 inhibitors have emerged as significant therapeutic agents in managing T2DM, particularly for their cardiovascular benefits. SGLT2 inhibitors not only lower blood glucose levels but also have been shown to cause significant reductions in the risks of major adverse cardiovascular events (MACE)in patients with T2DM and established cardiovascular disease (CVD) [30].

SGLT2 inhibitors have gained considerable attention for their therapeutic benefits in managing HF, particularly in patients with HFrEF and, more recently, in those with HFpEF. The EMPEROR-Reduced and DAPA-HF trials illustrate the effectiveness of SGLT2 inhibitors in HFrEF treatment, highlighting substantial reductions in the risk of hospitalization and cardiovascular death [31,32]. A meta-analysis further consolidates these findings, reporting lower rates of hospitalization for HF and cardiovascular complications associated with SGLT2 inhibitor therapy [33,34]. These benefits appear to result from various mechanisms, including improvements in myocardial energetics and reductions in fluid overload due to osmotic diuresis, thereby relieving cardiac workload [35].

In the context of HFpEF, the 2023 European Society of Cardiology (ESC) guidelines emphasize their role in improving cardiovascular outcomes, positioning SGLT2 inhibitors as a critical therapeutic option for patients with HFpEF and those with mildly reduced ejection fraction (HFmrEF) [27]. Relevant clinical trials, including EMPEROR-Preserved and DELIVER, have demonstrated that these agents significantly reduce the risk of hospitalization for HF and cardiovascular death, regardless of diabetes status [27].

Furthermore, SGLT2 inhibitors have been shown to reduce cardiac fibrosis, a recognized process in the pathophysiology of HF [36]. Their therapeutic efficacy is also driven by natriuretic and diuretic effects. By promoting glucose excretion and sodium loss through urine, these agents facilitate a reduction in extracellular fluid volume, leading to decreased body mass and improvement in HF symptoms [37,38,39]. In the meta-analysis of Cheong et al., the authors demonstrated a significant body mass reduction (mean BMI changes were −0.71 kg/m^2^ compared with placebo, *p* < 0.001) [40]. Moreover, Gaborit et al. suggested that these medications also lead to favorable changes in body composition specifically by reducing visceral fat [41]. The clinical benefits of SGLT2i, which appear rapidly, are likely driven by hemodynamic effects (osmotic diuresis, preload reduction) and shifts in myocardial energetics (ketone utilization) [35,42]. However, their long-term benefits may be substantially mediated by changes in body composition. The reduction in visceral and, critically, epicardial adipose tissue (EAT) is mechanistically significant. EAT is a source of pro-inflammatory cytokines (e.g., IL-6, TNF-α) that directly impair myocardial relaxation. Thus, SGLT2i-induced EAT reduction may be a crucial mechanism for improving diastolic function and mitigating the inflammatory substrate of HFpEF [43].

The contrasting data regarding the effects of SGLT2 inhibitors on muscle mass [44,45] warrant critical examination, as this is a major concern in potentially sarcopenic HF patients. These disparities may stem from several factors. Many studies use DEXA or BIA, which cannot easily distinguish intracellular from extracellular fluid. The potent osmotic diuresis from SGLT2-inhibitors causes a rapid reduction in extracellular fluid, which may be misinterpreted by these methods as a loss of lean body mass, when in fact it is a beneficial reduction in tissue edema, not a loss of contractile muscle protein [46]. Studies in younger T2DM patients may not reflect the reality of frail, elderly HF patients. The catabolic state of advanced HF, combined with caloric loss via glycosuria, could theoretically tip the balance towards muscle wasting in this specific, vulnerable population [47]. Short-term studies likely capture acute fluid shifts, whereas longer-term studies are needed to assess true changes in muscle homeostasis. Therefore, while the risk of true sarcopenia appears low and is likely outweighed by the benefits, there is an unmet need for studies using more precise methodology (e.g., MRI or CT) to confirm the long-term effects of SGLT2i on muscle quality and function (e.g., strength, CPET) in high-risk HF populations. Clinicians should remain cautious when initiating this therapy in patients with pre-existing frailty or sarcopenia.

Table 4 summarizes the results of the studies mentioned above.

In summary, the data from meta-analyses reveal consistent, multifaceted benefits of SGLT2 inhibitors. Clinically, beyond the established reduction in cardiovascular death risk, these agents consistently improve metabolic parameters, including HbA1c and blood pressure [37,38]. A critical finding is their robust impact on body composition. These drugs not only induce a moderate but consistent reduction in total body mass (mean −1.79 kg vs. placebo) but, more importantly, improve its composition [39,40]. However, while reductions in visceral fat are consistently reported [41], the claim that these benefits are achieved without compromising muscle mass [45] remains contentious. As discussed, methodological limitations of existing studies mean that the precise impact on muscle quality and function in frail HF populations is not definitively established, requiring further investigation.

## 6. Glucagon-like Peptide-1 Receptor (GLP-1R) Agonists

GLP-1R agonists are synthetic versions of the naturally occurring GLP-1 hormone, which plays pivotal roles in glucose metabolism, appetite regulation, and weight management. These pharmacological agents are designed to mimic native GLP-1 but have enhanced stability and bioactivity, thereby prolonging their therapeutic effects [48].

GLP-1 RAs have emerged as a relevant treatment in managing not only type T2DM but also CVD. Clinical evidence demonstrates that these agents offer cardiovascular benefits that extend beyond their glycemic control capabilities. GLP-1 RAs, such as semaglutide and liraglutide, have been shown to reduce MACE and improve various cardiovascular risk factors, including body mass, blood pressure, and lipid profiles [49,50,51].

The underlying mechanisms for these cardiovascular benefits are multifaceted. GLP-1 RAs exert protective effects on endothelial function and vascular smooth muscle cells, which can lead to improved vascular health and reduced atherosclerosis progression. Research suggests that GLP-1 receptor activation directly influences vascular function through pathways that enhance nitric oxide production and reduce oxidative stress [52,53]. Additionally, these peptides contribute to weight loss and improved insulin sensitivity, both of which are crucial in mitigating the risk of cardiovascular events in patients with obesity and diabetes [54].

In 2023, the ESC released comprehensive guidelines addressing the management of CVD in patients with diabetes, particularly focusing on the use of GLP-1 RAs. The guidelines reinforce the notion that GLP-1 RAs should be integral to the treatment strategies for patients with T2DM, especially those with concomitant atherosclerotic cardiovascular disease (ASCVD). The recommendation extends to patients who are already on other antidiabetic medications, wherein GLP-1 RAs can be incorporated to enhance cardiovascular protection without compromising glycemic control [55].

In the context of HF, recent meta-analyses highlight that GLP-1 RAs may reduce the risk of HF hospitalizations in patients with T2DM. Notably, the protective cardiovascular effects of GLP-1 RAs have been corroborated through various clinical trials, which report reductions in MACE and heart failure-related complications [56,57]. A meta-analysis by Ferreira et al. suggested that while GLP-1 RAs may be considered for reducing ischemic events in HFpEF patients with high atherosclerotic risk, they should be avoided in HFrEF patients pending further evidence [58]. Some studies demonstrated cardioprotective benefits of combination therapy with SGLT2 inhibitors [59].

GLP-1 RAs have shown promising effects in modifying body composition in patients with HF, particularly those with obesity and T2DM. These agents are recognized for their ability to induce significant weight loss especially in patients with HFpEF. In the study of Butler et al. treatment with semaglutide 2.4 mg improved symptoms, physical limitations and reduced inflammation in patients with HFpEF [60]. Kosiborod et al. revealed that the use of GLP-1 RAs contributes to an improvement in body composition parameters, which can be critical for maintaining functional capacity [61]. This profound improvement in HF-related symptoms and physical limitations may be partially mediated by the effects of GLP-1 RAs on key obesity-related comorbidities, such as obstructive sleep apnoea (OSA), which is highly prevalent in HFpEF. The significant weight loss driven by these agents can lead to favorable changes in regional fat distribution, including reductions in adipose tissue in the upper airways. This provides a direct mechanistic link between improved body composition, alleviation of OSA severity, and enhanced quality of life in this complex patient population [62]. In the STEP-HFpEF and STEP-HFpEF DM trials, semaglutide improved heart failure-related symptoms and physical limitations in participants with HFpEF. The profound weight loss seen in the STEP-HFpEF trials directly addresses the ‘obesity-phenotype’ of HFpEF. This benefit is likely mediated by a direct hemodynamic and mechanical unloading of the heart. Significant weight loss reduces total blood volume and systemic vascular resistance. The post hoc pooled, participant-level analysis of four randomized, placebo-controlled trials (SELECT, FLOW, STEP-HFpEF, and STEP-HFpEF DM) examined the effects of once-weekly subcutaneous semaglutide on HF events. Of the 22,282 trial participants, 3743 (16.8%) had a history of HFpEF (1914 semaglutide vs. 1829 placebo). In this cohort, semaglutide reduced the risk of the combined endpoint of cardiovascular death or worsening HF. This effect appeared to be driven by a reduction in worsening HF events, as the impact on cardiovascular death alone was not significant [63].

The implications of GLP-1 RAs for body mass composition and sarcopenia risk in patients with HF are increasingly recognized. On one hand, weight loss can mitigate the risk factors associated with sarcopenia, such as inflammation and metabolic dysregulation, commonly seen in HF patients [58]. On the other hand, the preservation of muscle mass and function is fundamental for promoting mobility and reducing frailty [64]. Osaka et al. demonstrated that treatment with semaglutide leads to favorable changes in appendicular skeletal muscle mass among older adults with T2DM and HF, signifying potential for GLP-1 RAs to address sarcopenia [65]. Further studies have supported these findings; one confirmed a beneficial effect of semaglutide on skeletal muscle mass [66], while Chun et al. revealed that treatment with semaglutide/cyanocobalamin led to increasing skeletal muscle mass [67]. Pandey et al. demonstrated an improvement in muscle quality in patients with metabolic syndrome during liraglutide therapy [68]. While studies suggest preservation of muscle quality (reduced myosteatosis) [68], the large-scale weight loss driven by semaglutide inevitably includes a reduction in absolute lean body mass, even if the proportion of fat loss is greater [69]. This raises a critical clinical concern for HF patients already on the cusp of sarcopenia. The key question is whether the functional benefits of reduced fat mass and improved mobility outweigh the potential risk of exacerbating frailty if absolute muscle mass falls below a critical threshold. Future research should focus on combining GLP-1 RA therapy with structured exercise interventions to maximize fat loss while actively preserving muscle mass. This highlights a critical knowledge gap, as the net benefit in frail, sarcopenic HFpEF patients is not yet established. The results of studies mentioned in this paragraph are presented in Table 5.

The data clearly differentiate the application of GLP-1 RAs based on HF phenotype. While SGLT2 inhibitors are first-line for HFpEF, GLP-1 RAs are a strong secondary consideration, especially if high atherosclerotic risk is also present [55,56,58]. Conversely, their use in HFrEF is currently not recommended pending further evidence [58]. A critical finding is the strong, positive impact of semaglutide in the specific subgroup of patients with HFpEF and obesity. In these patients, it significantly improves HF-related symptoms, physical limitations, exercise function, and quality of life [61,63]. These benefits are linked to a reduction in the combined endpoint of cardiovascular death or worsening HF events [63]. Evidence suggests these therapies improve body composition by significantly reducing fat mass. While some data indicate a preservation of muscle quality via reduced myosteatosis [66,68], this must be balanced against the clinical reality that significant absolute lean mass loss occurs. The net effect on functional capacity appears positive in HFpEF-obesity [61,63], but the long-term implications for frailty require careful monitoring and proactive management, such as combined exercise programs.

## 7. Glucagon-like Peptide-1 and Gastric Inhibitory Polypeptide (GLP-1/GIP) Receptor Agonists

GLP-1 and GIP receptor agonists represent a novel class of incretin-based therapies targeting metabolic disorders, including T2DM and obesity. These dual receptor agonists leverage the complementary actions of GLP-1 and GIP on glucose metabolism and appetite regulation. For instance, tirzepatide, a dual GLP-1/GIP receptor agonist, has shown significant improvements in glycemic control and weight loss compared to GLP-1 receptor agonists alone due to its enhanced mechanism of action [70,71].

The overarching benefit of these dual-action medications lies in their ability to reduce side effects typically associated with GLP-1 treatments, such as nausea. Research indicates that GIP receptor stimulation can mitigate the gastrointestinal discomfort caused by GLP-1 receptor activation, making these medications more tolerable for patients [71]. Furthermore, studies suggest that dual agonists may lead to improvements in insulin sensitivity and beta-cell function relative to single-agent therapies [72].

The dual action of tirzepatide promotes improved glycemic control and body mass reduction, both of which are critical in reducing mechanical stress on the heart. Studies suggest that tirzepatide leads to reductions in HF hospitalizations, likely due to enhanced left ventricular performance and improved metabolic profiles [73,74]. Specifically, the SURPASS-2 clinical program indicated that patients treated with tirzepatide exhibited better cardiovascular outcomes relative to those receiving only standard therapies to manage diabetes [74]. In another study, the authors showed reduction in the risk of HF and MACE, stroke, chronic kidney disease (CKD), and coronary artery disease (CAD) in patients without DM during tirzepatide therapy [75]. These findings are consistent with those of other researchers [76]. The mechanisms through which tirzepatide exerts these effects include improved insulin sensitivity and modulation of lipid metabolism, which are beneficial for patients at-risk of HF [77,78].

Research has also highlighted the importance of body composition changes associated with tirzepatide treatment. Trials report not only significant weight loss but also favorable changes in fat distribution, including reductions in visceral fat that further attenuate cardiovascular risks associated with obesity [78,79,80]. Moreover, Kramer et al. demonstrated that tirzepatide therapy in obesity-related HFpEF led to reduced left ventricular (LV) mass and paracardiac adipose tissue as compared with placebo, and the change in LV mass paralleled weight loss [81]. This reduction in left ventricular mass and paracardiac adipose tissue is a critical pathophysiological link. It suggests tirzepatide directly remodels the heart’s immediate environment by reducing lipotoxicity and mechanical constraint, offering a direct pathway to improved diastolic function that parallels, and is likely driven by systemic weight loss. These physiologic changes may contribute to the reduction in heart failure events seen in the main SUMMIT trial [80]. Table 6 summarizes the results of the studies mentioned above.

A primary finding is its consistent superiority over semaglutide, demonstrating significantly greater reductions in both HbA1c and total body weight [74]. Tirzepatide shows particular promise for patients with HFpEF and obesity. In this population, it reduces the risk of a composite of cardiovascular death or worsening HF and improves health status [76,80]. Mechanistically, this is supported by evidence of reduced left ventricular mass and paracardiac adipose tissue, changes that parallel weight loss [81]. Beyond HFpEF, cohort studies associate tirzepatide use with lower risks of HF exacerbation, all-cause mortality, MACE, and major adverse kidney events [75,76]. The superior weight loss is characterized by a greater reduction in fat mass compared to semaglutide [77,78]. Furthermore, tirzepatide favorably modifies atherogenic lipoprotein profiles by reducing levels of apoC-III, apoB, and atherogenic lipoprotein particles, suggesting a direct mechanism for reducing cardiovascular risk [79].

While the preceding sections detailed each drug class individually, Table 7 provides a comparative summary of their core mechanistic differences, established HF indications, and specific impacts on body composition.

## 8. Conclusions

**Figure 3:**
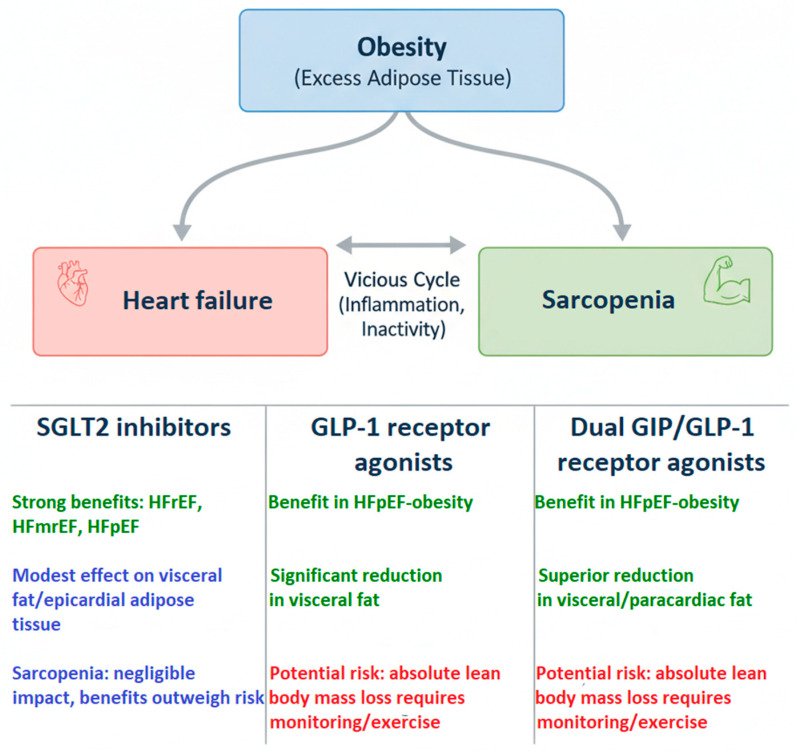
Conceptual framework of pharmacological interventions on body composition in heart failure.

In conclusion, the management of HF has entered a metabolic era, where the distinction between reducing “weight” and improving “body composition” is critical. As this review has analyzed, SGLT2 inhibitors, GLP-1 receptor agonists, and dual GIP/GLP-1 receptor agonists offer profound cardiovascular benefits, but their mechanisms and risks related to body composition differ significantly. Integrating these therapies requires a nuanced approach, particularly in patients with obesity or sarcopenic obesity, to enhance symptom control and long-term prognosis. A conceptual framework summarizing these complex, multifactorial interactions is presented in Figure 3.

However, to fully leverage these advances, a forward-looking perspective is crucial. A critical need exists for further research elucidating the precise mechanisms by which these therapies modulate body composition in diverse HF phenotypes, especially their differential effects on adipose tissue versus lean muscle mass. This endeavor necessitates the identification and validation of novel biomarkers and imaging modalities to accurately track longitudinal changes in body composition. Relying on traditional metrics such as BMI alone is misleading, as it fails to distinguish between metabolically active adipose tissue and protective lean muscle mass—a distinction that is central to the pathophysiology of HF and the risk of sarcopenia. While more informative anthropometric measures, such as waist circumference or waist-to-height ratio, can provide a better indicator of at-risk individuals, the field must ultimately move towards advanced, non-invasive biomarkers (such as specific myokines, adipokines, or advanced imaging) to go beyond simple anthropometry.

Furthermore, prioritizing future clinical trials designed to directly assess these metabolic–cardiac interactions is paramount. Such trials must clarify how therapeutic-induced changes in body composition (e.g., reduction in visceral fat, preservation of muscle) translate directly into improved cardiac structure and function, such as ventricular remodeling and diastolic performance. Addressing these research priorities will be essential to truly personalize metabolic interventions in the complex, heterogeneous HF population.

## Figures and Tables

**Table 1:** Diagnostic methods for sarcopenia and their application in the HF population.

Diagnostic Method	Parameter Measured	Advantages	Limitations and Application in the HF Population
DXA (Dual-energy X-ray Absorptiometry)	Muscle quantity (Appendicular Lean Mass, ALM)	Gold standard for body composition assessment; precise.	Availability, cost. Results can be falsely elevated by fluid overload (edema), which is common in HF, masking lean mass loss.
BIA (Bioelectrical Impedance Analysis)	Muscle quantity (Skeletal Muscle Mass, SMM)	Portable, inexpensive, fast, non-invasive.	Highly dependent on hydration status. In HF patients, especially during decompensation, results are often unreliable due to edema and fluid shifts. Requires specific equations validated for HF.
Handgrip Strength	Muscle strength	Inexpensive, fast, portable. Strong prognostic predictor. Recognized by EWGSOP2 as the primary screening indicator.	Easy to implement. Should be a routine screening test in HF patients to identify sarcopenia risk.
Muscle Ultrasound	Muscle quality and quantity (e.g., quadriceps thickness, echogenicity)	Portable (Point-of-Care), non-invasive. Unaffected by edema when assessing muscle thickness. Allows assessment of quality (e.g., fat infiltration).	Requires standardization and trained operators. Increasingly promising for bedside assessment in HF patients.
Computed Tomography (CT)/Magnetic Resonance Imaging (MRI)	Muscle quantity and quality (cross-sectional area, fat infiltration)	Highly precise; allows for accurate assessment of muscle fat infiltration (myosteatosis).	High cost, radiation exposure (CT), limited availability. Reserved mainly for research purposes, not routine HF diagnostics.

**Table 2:** The causes of sarcopenia in HFrEF. LPS—lipopolysaccharide, TNF-α—tumor necrosis factor alpha, ROS—reactive oxygen species.

The Causes of Sarcopenia in HFrEF
increase in inflammatory parameters
LPS stimulates the production of TNF-α
muscle atrophy due to disuse
autophagy associated with ROS, damaged proteins, damaged organelles, and hypoxia
activation of protein degradation via the ubiquitin-proteasome pathway in skeletal muscles
insulin resistance
changes in the growth hormone/insulin-like growth factor axis
growth hormone insensitivity
sympathetic overactivity
increased myostatin levels

**Table 3:** The causes of sarcopenia in HFmrEF and HFpEF.

The Causes of Sarcopenia in HFmrEF and HFpEF
inflammation
abnormal intramuscular adipose tissue
oxidative stress
insulin resistance—suppressed growth hormone secretion and decreased insulin-like growth factor I

**Table 4:** Characteristics of included studies.

Study (Year)	Number of Patients	Design	Findings
Yankah R. et al. (2024) [37]	55.501	Meta-analysis	Reduction in risk of cardiovascular death, reductions in blood pressure and body mass.
Davies M. et al. (2022) [38]	2.313	Post hoc analysis	Reductions in HBA1c, body mass, and systolic blood pressure in patients with CVD or CVD risk factors.
Pan R. et al. (2022) [39]	1.430	Meta-analysis	Improving body composition in T2DM, specifically through reductions in key anthropometric measures (body mass, BMI, waist circumference) and adiposity (visceral, subcutaneous, and total fat mass).
Cheong A. et al. (2022) [40]	98.497	Meta-analysis	A mean weight reduction of −1.79 kg (95% CI: −1.93 to −1.66, *p* < 0.001) compared with placebo was observed across diabetes status, duration of follow-up, various comorbidities, and all SGLT drug types.
Gaborit B. et al. (2021) [41]	80	Prospective interventional study	Empagliflozin selectively reduced liver fat content (−27 ± 23 vs. −2 ± 24%, *p* = 0.0005) and visceral fat (−7.8% [−15.3;−5.6] vs. −0.1% [−1.1;6.5], *p* = 0.043), with no effect observed on myocardial or epicardial fat.
Jaiswal A. et al. (2023) [42]	15.989	Meta-analysis	Shifting of fatty acids to ketone bodies as the substrate for myocardial energy generation
Yabe et al. (2023) [45]	129	Randomized, double-blind, placebo-controlled trial	Empagliflozin improved glucose control and reduced body weight without compromising muscle mass and strength in older adults with T2DM.

**Table 5:** Characteristics of included studies.

Study (Year)	Number of Patients	Design	Findings
Obata S. et al. (2023) [56]	8.965	Meta-analysis	Reduction MACE in T2DM patients with prior HF compared with the placebo group
Neuen B. et al. (2023) [57]	1.743	Meta-analysis	Cardiovascular and kidney benefits of GLP-1 receptor agonists in T2DM patients regardless of SGLT2 inhibitor use
Ferreira J. et al. (2023) [58]	11.430	Meta-analysis	GLP-1 RAs should be considered for improving cardiovascular outcomes in T2DM patients with atherosclerotic risk but no HF. In T2DM patients with HFpEF and high atherosclerotic risk, they may be considered as a second-line agent after an SGLT2 inhibitor. They should, however, be avoided in HFrEF patients until further evidence is available.
Marfella R. et al. (2024) [59]	537	Observational cohort study	The incidence of MACE was lower in the combination therapy group compared with patients receiving either SGLT2i or GLP-1RA monotherapy
Butler J. et al. (2023) [60]	529	Randomized, double-blind, placebo-controlled trial	In patients with HFpEF and obesity, semaglutide 2.4 mg improved symptoms, physical limitations, and exercise function, while also reducing inflammation and body mass. These benefits were observed to a similar extent across all LVEF categories.
Kosiborod M. et al. (2024) [61]	529	Analysis	Benefits of semaglutide extended to all key summary and individual KCCQ domains
Kosiborod M. et al. (2024) [63]	22.282	Randomized controlled trial	In patients with HFpEF, semaglutide reduced the risk of the combined endpoint of cardiovascular death or worsening HF events, and worsening HF events alone, whereas its effect on cardiovascular death alone was not significant.
Nelson L. et al. (2024) [66]	241	Retrospective study	Semaglutide may preserve muscle quality during weight loss, as muscle attenuation remained stable despite a slight reduction in muscle mass. Conversely, in patients who gained weight, the decline in attenuation (myosteatosis) and increased intermuscular fat were likely attributable to overall fat accumulation, not a direct drug effect.
Chun E. et al. (2025) [67]	94	Retrospective study	Semaglutide/ Cyanocobalamin led to significant weight loss, with fat mass reduction and a proportional increase in skeletal muscle mass, suggesting an improvement in body composition despite some lean mass loss.
Pandey A. et al. (2024) [68]	128	Randomized controlled trial	Liraglutide reduced muscle fat infiltration but did not significantly affect overall muscle volume, suggesting a potential improvement in muscle quality rather than quantity.

**Table 6:** Characteristics of included studies.

Study (Year)	Number of Patients	Design	Findings
Frias J. et al. (2021) [74]	1.879	Randomized, double-blind, placebo-controlled trial	All tirzepatide doses showed significantly greater HbA1c reductions than semaglutide. Tirzepatide demonstrated superior weight loss across all doses compared to semaglutide.
Augusto S. et al. (2025) [75]	897	Review	Tirzepatide presents a promising therapeutic option for managing HF, with significant metabolic and cardiovascular benefits. Untreated patients were at higher risk of incident acute HF and MACE, stroke, CKD, and CAD.
Lin Y. et al. (2025) [76]	14.154	Retrospective cohort study	Tirzepatide use was associated with significantly lower risks of the primary composite outcome of HF exacerbation and all-cause mortality, as well as reductions in major adverse cardiovascular events and major adverse kidney events.
Heise T. et al. (2023) [77]	117	Randomized, double-blind, placebo-controlled trial	Tirzepatide led to greater reductions in body mass and fat mass than placebo and semaglutide; however, while both active drugs reduced appetite significantly more than placebo, they did not differ from each other on this measure.
Roux C. et al. (2023) [78]	11.430	Meta-analysis	Tirzepatide (particularly at 15 mg) may offer superior weight loss efficacy compared to semaglutide 2.4 mg, with a similar tolerability profile.
Wilson J. et al. (2020) [79]	316	Randomized, double-blind, placebo-controlled trial	Tirzepatide treatment dose-dependently decreased levels of apolipoprotein C-III (apoC-III) and apolipoprotein B (apoB) and the number of large triglyceride-rich lipoproteins (TRLP) and small low-density lipoprotein particles (LDLP), suggesting a net improvement in atherogenic lipoprotein profile.
Packer M. et al. (2024) [80]	731	Randomized, double-blind, placebo-controlled trial	In patients with HFpEF and obesity, tirzepatide led to a lower risk of the composite of death from cardiovascular causes or worsening HF than placebo. Additionally, it improved health status.
Kramer CM et al. (2024) [81]	175	Randomized, double-blind, placebo-controlled trial	In obesity-related HFpEF, tirzepatide therapy reduced LV mass and paracardiac adipose tissue compared with placebo. Furthermore, the reduction in LV mass paralleled weight loss.

**Table 7:** Summary of mechanistic differences and body composition effects of modern metabolic therapies in heart failure.

Feature	SGLT2 Inhibitors	GLP-1 Receptor Agonists	GIP/GLP-1 Receptor Agonists
Primary mechanism	Inhibits glucose/sodium reabsorption in the proximal tubule.	Activates GLP-1 receptors.	Activates both GIP and GLP-1 receptors.
HF indication	HFrEF, HFmrEF and HFpEF	HFpEF with obesity	HFpEF with obesity
Body weight loss	Modest (~2–3 kg)	Significant (~15–17%)	Very significant (~20%+)
Effect on fat mass	Reduces visceral and epicardial fat.	Strong reduction in total fat mass	Superior reduction in total fat mass. Reduces paracardiac fat.
Effect on lean mass	Contentious. Likely reduction in fluid/edema misinterpreted as lean mass loss. Risk of true sarcopenia considered low, but needs study.	Absolute lean mass is lost alongside fat mass. Muscle quality (myosteatosis) may be preserved or improved.	Absolute lean mass is lost, but fat loss is preferential
Key cardiovascular mechanism	Hemodynamic (diuresis, preload reduction), metabolic (ketone shift, epicardial adipose tissue reduction)	Anti-inflammatory, hemodynamic (unloading via weight loss), atherosclerotic risk reduction	Superior weight loss (hemodynamic), adipose tissue remodeling (paracardiac fat), improved atherogenic lipids
Primary concern	Potential fluid/lean mass ambiguity	Risk of exacerbating sarcopenia due to large absolute lean mass loss, gastrointestinal side effects	Risk of exacerbating sarcopenia due to large absolute lean mass loss, gastrointestinal side effects

## Data Availability

No new data were created or analyzed in this study. Data sharing is not applicable to this article.
